# A Review of Pediatric Orthopedic Disorders: Diagnosis and Treatment Updates

**DOI:** 10.7759/cureus.101486

**Published:** 2026-01-13

**Authors:** Sanjay P, Ravi Diwakar, Subeg Singh, Rahul H Gujarathi, Binita J Purohit, Kalpana Patni

**Affiliations:** 1 Sports Medicine, Saveetha Institute of Medical and Technical Sciences, Chennai, IND; 2 Orthopaedics, Virendra Kumar Sachlecha Government Medical College, Neemuch, IND; 3 Orthopaedics, Guru Gobind Singh Medical College and Hospital, Faridkot, IND; 4 Paediatrics, Bharati Vidyapeeth Ayurved College, Pune, IND; 5 Anatomy, Dr N D Desai Faculty of Medical Science and Research, Dharmsinh Desai University, Nadiad, IND; 6 Kaumarbhritya (Ayurveda Pediatrics), Institute of Medical Sciences, Banaras Hindu University, Varanasi, IND

**Keywords:** clubfoot, developmental dysplasia, fractures, pediatric orthopaedics, scoliosis

## Abstract

Orthopedic disorders in pediatrics have a broad range of congenital and acquired musculoskeletal ailments, which significantly affect development, mobility, and quality of life in children. This review summarizes the existing evidence in the management of major disorders, such as developmental dysplasia of the hip, clubfoot, scoliosis, slipped capital femoral epiphysis, fractures, and neuromuscular syndromes. The relevant studies were identified by a thorough search in PubMed, EMBASE, Cochrane Library databases, recent trials, and consensus guidelines. Technological improvements such as ultrasonographic screening, the Ponseti method, producing a success rate of more than 90%, growth modulation implants, and motorized limb lengthening systems have enhanced the outcome and minimized the surgical load. Nevertheless, there are still issues such as inconsistency in the protocols, a lack of comparative research, and inequality in access to special care. New technologies, such as three-dimensional imaging, patient-specific instrumentation, and artificial intelligence-based predictive models, have the potential to be more precise but will need tight scrutiny of safety and cost-effectiveness. In future studies, multicenter trials, standardized outcomes with patient-reported outcome measures, and consensus guidelines to standardize care should be given priority. Continued investment in training, infrastructure, and international collaboration will be needed to ensure that innovations are fairly introduced and can be measured as leading to measurable improvement in musculoskeletal health and functioning of children across the world.

## Introduction and background

Orthopaedic disorders in children are a wide range of congenital and acquired musculoskeletal disorders, which have a major impact on the growth, mobility, and quality of life of the children [1]. These disorders are spread between the relatively benign, self-limiting disorders and the severe deformities and chronic diseases that need a multidisciplinary approach to their management [2]. The development of early detection, imaging techniques, and surgical procedures has led to better results, and studies have found a 60% drop in the rate of surgical interventions when early screening is used in some of the conditions [3]. However, the issue of unequal access to special care is still a problem in most areas. The overall impact of musculoskeletal disease in children is high, and in isolation, congenital anomalies contribute a significant percentage of the burden of childhood disability and healthcare utilisation [4]. It is important to diagnose and intervene as early as possible because, otherwise, it may lead to progressive deformity and loss of functionality in children and their families and have psychosocial implications [5].

The most frequently met conditions are developmental dysplasia of the hip (DDH), clubfoot, scoliosis, slipped capital femoral epiphysis (SCFE), Legg-Calve-Perthes disease, and a set of various fractures and bone infections [6]. Both of them have unique diagnostic and treatment issues that have changed with time due to technology and evidence-based practices [7]. For example, there is now mass ultrasonographic screening of DDH and perfecting of the Ponseti technique of clubfoot treatment, and the early treatment has changed the landscape, and subsequent invasive treatment is no longer necessary, but there is still controversy whether bracing should be performed long enough to prevent relapse [8]. On the same note, growth modulation devices and magnetically controlled growing rods have increased the range of choices in the treatment of early-onset scoliosis [9].

**Figure 1:**
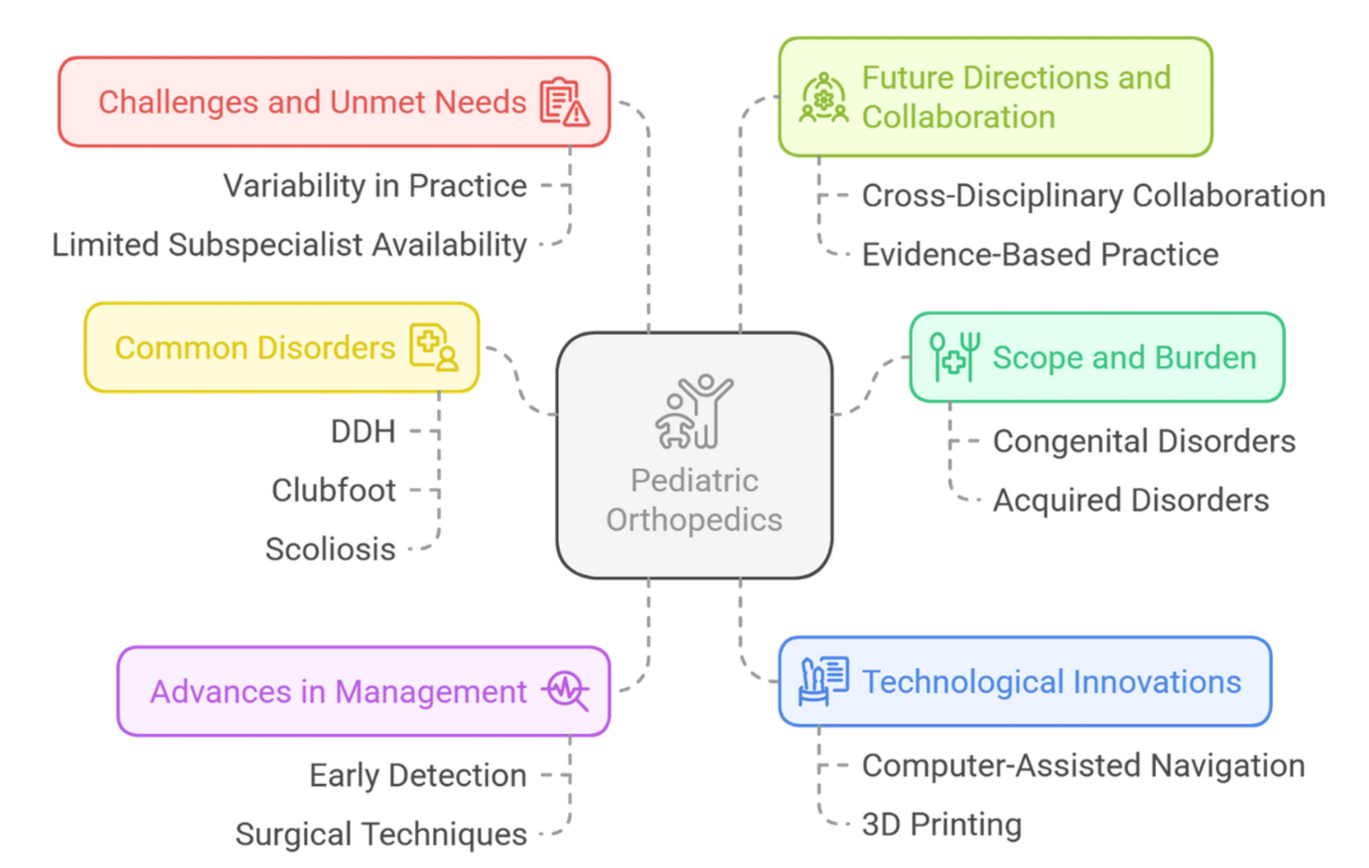
Conceptual Overview of Pediatric Orthopedic Disorders Image Credit: Sanjay P

Fractures in children continue to be a common reason for orthopaedic referral, and special attention is given to the growth plates and the ability of immature bone to remodel [10]. Physeal injuries should be correctly diagnosed because failure to diagnose or mis-treat the injury may result in growth disturbances and deformities [11]. The recent advances in imaging, such as low-dose biplanar radiography and 3D reconstructions, have increased the precision in the classification of fractures and the planning of the surgical procedures, but the question of the cumulative effect of radiation to the young patients has not been completely addressed [12]. Such as the scope of the diseases, prevalent diseases, development in management, novel technologies, unmet needs, and areas of collaboration (Figure 1).

The infections of the bones and joints, especially acute hematogenous osteomyelitis and septic arthritis, remain a challenge to manage [3]. Advances in microbiological diagnostics have made it possible to identify pathogens earlier, and the problem of antibiotic resistance and uncharacteristic presentations is still an obstacle [10]. The trend toward shorter intravenous antibiotic treatment and early switch to oral therapy is the result of developing a consensus that aims at minimising hospitalisation without affecting outcomes, but well-designed comparative studies in pediatric patients are scarce [13].

Treatment of neuromuscular diseases, particularly cerebral palsy, poses a complex challenge that involves medical, surgical, and rehabilitative interventions that involve treatment of contractures, hip dislocation, and gait disorders [14]. Current guidelines focus on early monitoring and treatment to avoid sequential deformities and maximise performance. In the same way, metabolic and genetic bone diseases such as osteogenesis imperfecta have also seen improvement in their treatment, such as the use of bisphosphonate regimens that enhance the densities of the bones and lower the rates of fractures by up to 50% [5]. Technology innovation has transformed the field of pediatric orthopaedics, and the use of computer-assisted navigation, patient-specific instrumentation, and three-dimensional printing has become common in the field of complex reconstruction [15]. The modalities can be more precise and tailored, but their cost-effectiveness and long-term outcomes are subject to further research, particularly in low-resource environments [7]. There are also concomitant developments in biologics, gene therapy, and tissue engineering, which are opening up new possibilities to previously untreatable diseases [16]. However, these new treatments have to undergo stringent clinical trials that are well-designed to determine safety and efficacy [11].

Nonetheless, the problems of early diagnosis and equal access to quality care remain in place even after remarkable improvements [17]. The practice patterns are variable, there is no standardised protocol, and there are few pediatric orthopaedic subspecialists, which still impede the achievement of optimal outcomes [3]. The lack of high-level evidence in the form of randomised trials has led to the continuity of arguments on the best procedures, and there is an immediate need to promote multicenter collaborations and standard outcome reporting [9]. In that regard, it is necessary to synthesise the contemporary knowledge in order to inform the evidence-based practice and conduct additional studies [13]. Interdisciplinary cooperation, with radiology, infectious disease, rehabilitation, and genetics being a few examples, is still a core pillar of complete care [15].

To provide a broader framework for readers unfamiliar with the discipline, pediatric orthopaedics encompasses not only the management of specific musculoskeletal conditions but also the overarching goal of maintaining normal growth, alignment, and function throughout childhood. The disorders highlighted in this review, namely, DDH, clubfoot, scoliosis, SCFE, fractures, neuromuscular disorders, and infections, were selected because they represent the most prevalent, clinically impactful, and conceptually illustrative conditions encountered in practice. By outlining their epidemiology, diagnostic principles, and treatment advances, the introduction aims to make the review accessible both to trainees and to readers who may be less familiar with the field.

The review aims to give a current synthesis of diagnostic procedures and treatment plans of common pediatric orthopaedic disorders, to review new technological and therapeutic advances that affect clinical practice, and to evaluate current evidence of these approaches. This review also seeks to point out knowledge gaps and priorities of future research to enhance the care and outcomes for children with musculoskeletal conditions and areas where existing recommendations are controversial or lack substantial comparative evidence.

Methods

This article was conducted as a narrative review. A targeted literature search was performed in PubMed, EMBASE, and the Cochrane Library using combinations of the following terms: “pediatric orthopaedics”, “developmental dysplasia of the hip”, “clubfoot”, “scoliosis”, “pediatric fractures”, “slipped capital femoral epiphysis”, “cerebral palsy”, and “osteomyelitis”. Recent guidelines, consensus statements, and high-impact studies published within the last 10-12 years were prioritised. Studies were selected based on clinical relevance, contribution to diagnostic or therapeutic understanding, and applicability to contemporary practice. As this is a narrative review, no formal risk-of-bias assessment or evidence weighting was undertaken. Only English-language articles focusing on pediatric populations and addressing diagnosis, management, or treatment outcomes were considered for inclusion.

## Review

Developmental dysplasia of the hip (DDH)

The DDH is a range of conditions that are caused by the insufficient coverage of the acetabulum of the femoral head, which leads to instability or dislocation in the case of no treatment [18]. The incidence has been reported as either 1-20 out of 1,000 live births, depending on ethnicity, geography, and screening practices [19]. The most conspicuous perinatal risk factor is the breech presentation, which elevates the instability in the hip by a factor of almost 10 as compared to the cephalic deliveries [3]. Other risk factors are female sex, family history of DDH, oligohydramnios, and being a firstborn child, which indicate mechanical or hormonal effects on the development of joints [2].

The neonatal clinical examination is also the basic one, and the main method of detecting instability is the Barlow and Ortolani maneuvers [11]. Nevertheless, their sensitivity decreases after the initial weeks, and this is why ultrasonographic screening is widely used [12]. The most validated sonographic system is the Graf classification, which classifies hips according to the alpha and beta angles. Type I hips are normal, whereas type IIa hips are immature in infants below three months and normally will mature spontaneously. Type IIb hips have late ossification that has to be treated in case it lasts more than three months. Type III hips are subluxated and partially dislocated, and type IV hips are fully dislocated [5].

The development of ultrasonography has underlined the importance of dynamic assessment at four to six weeks with better reproducibility and decreased overtreatment [20]. High-risk infant selective screening is prevalent, and universal screening has minimized late dislocations. As an example, in Austria, the incidence of late detection decreased by 1.5 to less than 0.2 per 1,000 live births following the introduction of universal ultrasonography [21]. Nevertheless, there are still issues related to overtreatment and augmented spending on healthcare, which explains the necessity of balanced risk stratification. The Pavlik harness is the treatment of choice for reducible dysplastic hips that are diagnosed before six months [14]. This dynamic brace keeps the flexion and abduction, and it encourages the acetabular remodeling and decreases the pressure of the femoral head [8]. Early treatment before eight weeks and close follow-up increases the success and is over 90% in stable hips at one-year follow-up [1]. It is advised that ultrasonographic confirmation of concentric reduction be done within three weeks [22]. Avascular necrosis is a complication that happens in 1-5% of cases, especially when excessive abduction is used [4]. Recent research indicates that there is no added value in the use of the harness after 12 weeks and that it may even lead to more skin issues, but the evidence is of low quality.

Surgical management is required when harness treatment fails or is late to start [9]. In infants within 6-18 months, general anesthesia with closed reduction is standard, and arthrography should be used to confirm the reduction and to evaluate interposition of soft tissues [23]. Open reduction through an anterior approach in irreducible hips enables the removal of such obstacles as the pulvinar or hypertrophic ligamentum teres [12]. Up to 20% of cases are left with residual dysplasia, especially in hips that have been treated after six months [3]. Pelvic osteotomies may be necessary in older children or those with ongoing acetabular deficiency to enhance coverage [14]. Salter innominate osteotomy restores the acetabulum in an anterior and lateral orientation, and the periacetabular osteotomy (PAO) gives the multiplanar correction and maintains the pelvic stability [24]. PAO has demonstrated positive results with an approximate 80-90% survival rate without the progression of osteoarthritis at 15-20 years [15]. However, comparative data of PAO with other pelvic osteotomies in adolescents are limited to retrospective studies, and therefore, prospective trials are needed.

MRI and 3D CT enhance the preoperative planning and postoperative evaluation since they are able to visualize labral cartilage and the sphericity of the femoral heads [13]. MRI is of special value when evaluating the quality of reduction and in forecasting residual dysplasia [6]. The radiographs are normally performed at 3-, 6-, and 12-month intervals following reduction and yearly until skeletal maturity [10]. Disparities in early diagnosis remain a significant global issue. In the low- and middle-income countries, the presentation is usually delayed, which often requires complex reconstructive surgery [2]. Standardized training and awareness among the populace have been encouraged through programs such as the International Hip Dysplasia Institute to deal with these disparities [25]. It is necessary to conduct a study in the future that will help optimize screening strategy, clarify the minimum effective bracing time, and establish agreement regarding the surgical indication to limit variation in care.

Clubfoot (congenital talipes equinovarus)

Clubfoot is a developmental malalignment condition that has complex multiplanar malalignment, and when not treated, it causes a lot of disability and social stigma [26]. Even though its etiology is not fully understood, there is a likelihood that both genetic and environmental factors tend to interact, thereby preventing normal foot development [12]. With recent improvements in treatment in the past decades, prognosis has significantly improved, especially with the widespread use of the Ponseti method, which has substituted more invasive surgical procedures as the standard of care [27]. It is also important to start therapy at an early age, since at this stage the soft tissue is soft, which allows correction with minimal intervention gradually [1].

Although the overall success rates are high, clubfoot treatment is a demanding process that needs detailed care and a long-term follow-up to avoid a relapse [28]. The bracing adherence has become one of the most important factors that contribute to the long-term correction, and it is still difficult to achieve in many families [3]. Non-adherence not only raises the possibility of relapse but also frequently requires further procedures that may affect the functioning [7]. Although promising results have been obtained in terms of compliance with dynamic bracing technologies, strong comparative studies are required to define their relative effectiveness and guide the development of guidelines [29].

Atypical or complex clubfoot represents a distinct and more challenging subgroup characterized by a smaller and stiffer foot, severe equinus, deep medial and plantar creases, rigid cavus, and pronounced adduction [14]. These deformities often respond poorly to standard Ponseti casting and therefore require modified techniques, including gentler manipulation, limited abduction during casting, and more frequent cast changes [6]. Atypical cases also demonstrate higher relapse rates and reduced bracing tolerance, making close follow-up essential to prevent early recurrence. Although modified Ponseti protocols have shown favorable outcomes in several clinical series, high-quality comparative studies remain lacking, and there is currently no universal consensus on the optimal management of atypical presentations [20].

Tendon transfers and adjunct procedures might be significant in the re-establishment of dynamic muscle balance in persistent or refractory deformities [10]. Such interventions are, however, to be considered with caution in the light of overcorrection, stiffness, and early degenerative changes [15]. Imaging is mostly used in unusual or syndromic cases in which clinical evaluation is not adequate [5]. MRI and ultrasound provide useful data regarding the joint congruity and constraints of the soft tissues, particularly in cases where surgery planning is required [8].

Access to early diagnosis and universal protocols of care is an ongoing issue in the world [2]. The incomplete treatment courses and delays in the initiation of the treatment still contribute to preventable disability in low-resource settings. Such efforts as the Global Clubfoot Initiative have shown that a massive expansion of Ponseti training and support systems can have a transformative effect on the outcomes, yet the success of such programs is dependent on the continuous investment and the development of local capacity [14]. The future research and policy priorities are the critical assessment of the alternative bracing techniques, international consensus on the treatment duration, and long-term follow-up of the functional outcomes in adulthood [19]. Table 1 presents the key points about clubfoot that include its causes, methods of treatment, outcomes, and priorities for the future.

**Table 1:** Overview of Clubfoot: Etiology, Management, Outcomes, and Implementation Challenges

Aspect	Details	Treatment and Bracing	Outcomes	Future Directions	Reference
Definition	Congenital deformity with forefoot adduction, hindfoot varus, midfoot cavus, ankle equinus	Ponseti method: serial casting (5–7 casts), Achilles tenotomy in >80%	>90% achieve plantigrade, pain-free feet at 10 years	Multicenter trials comparing bracing protocols, adjunct therapies for soft tissue remodeling	[30]
Epidemiology	Occurs in ~1–2 per 1,000 live births; ~25% recurrence risk among first-degree relatives	Bracing: full-time 3 months, nighttime to ≥4 years; dynamic braces under investigation	Relapse rates 20–30% beyond adolescence; non-adherence linked to recurrence	Longitudinal studies of functional outcomes into adulthood	[7]
Etiology	Multifactorial genetic and environmental factors	Tibialis anterior tendon transfer for recurrent deformities	Good results in reducing relapse when the tendon transfer is performed	Sustainability of training programs in low-resource settings depends on investment and infrastructure	[12]
Imaging	Not routinely required in idiopathic cases; indicated for atypical, rigid, or syndromic presentations	Ultrasound: navicular-talus alignment; MRI for complex deformities	Imaging assists in pre-surgical planning for complex cases	Development of standardized imaging protocols	[31]
Historical Comparison	Surgical posteromedial release is associated with ~50% stiffness, overcorrection, and early degenerative arthritis	Now largely replaced by the Ponseti technique	Lower complication and relapse rates compared to historical surgical release	Research on optimizing duration and methods of bracing to further reduce relapse	[15]
Challenges & Implementation	Access to care varies worldwide; incomplete casting and delayed treatment in resource-limited settings often necessitate surgical release	The Global Clubfoot Initiative has improved training and outcomes globally	Improved early outcomes where programs are implemented	Continued investment in education, capacity-building, and outcome tracking to sustain progress	[29]

Pediatric fractures: principles and pitfalls

Fractures in children comprise up to 25% of all childhood injuries that are brought to the emergency departments [9]. The growing bone has some unique features, such as a thicker periosteum, increased remodeling ability, and growth plates that differentiate pediatric fractures and adult trauma [32]. During growth, angular deformities in the plane of motion of the joint, e.g., sagittal angulation in the distal radius, reliably remodel, but rotational deformities do not and usually need anatomic reduction to avoid long-term functional impairment [33]. Physeal injuries require special vigilance. The Salter-Harris classification defines five patterns of injury. Type I injuries entail a total detachment of the physis without fracture of the bone. Type II fractures penetrate the physis and the metaphysis, forming a metaphyseal fragment. Type III fractures extend the epiphysis and physis to the articular surface. Type IV injuries extend beyond the metaphysis, physis, and epiphysis, which interfere with the cartilage in the joints. Type V injuries are crush injuries of the physis and bear the greatest potential of growth arrest [34]. The risk of growth arrest is different between types and sites, with a 1-3% risk in type I and II fractures, a 25% risk in type IV injuries, and a 50% risk in distal femoral physeal fractures [22]. Early diagnosis and anatomic reduction of higher-grade physeal injuries are key to reducing the occurrence of permanent deformity, and there is a general agreement that superior fixation methods have yet to be established due to the absence of high-quality comparative studies.

Flexible intramedullary nailing has now come to be the procedure of choice in most of the diaphyseal fractures of the femur and forearm in school-going children [13]. The union rates are more than 95% in meta-analyses, and the malunion rates, as well as the time of mobilization, are lower than in traction or spica casting [23]. The complication rates, such as implant irritation and entry site infections, are reported, on average, at less than 10%; thus, this technique can be considered a safe alternative in properly chosen patients. Recent randomized trials indicate the support of shorter immobilization and functional bracing in stable fractures. In an example, distal radius fracture removable splints applied during two to three weeks have shown the same healing and greater patient satisfaction as compared to traditional casting [6,24]. Nevertheless, the effect of the early mobilization protocols on the physeal growth and remodeling in the long term is the subject of further investigation.

High-resolution imaging, such as 3D reconstructions and low-dose biplanar radiography, has enhanced the capacity to assess the complex intra-articular or physeal injury and design surgical fixation [5]. MRI is particularly useful in the identification of the occult physeal separations or ligamentous injury [8]. Although these are the advantages, cumulative radiation exposure should be reduced as much as possible [10]. The consensus guidelines advise careful application of CT and repeating radiographs, including use with younger children, to minimize radiologic exposure to radiation throughout their lifetime. Family-centered care with explicit advice on the anticipated healing period, limitations on activities, and the indicators of complications that may arise has been observed to enhance adherence, as well as decrease anxiety [14].

**Figure 2:**
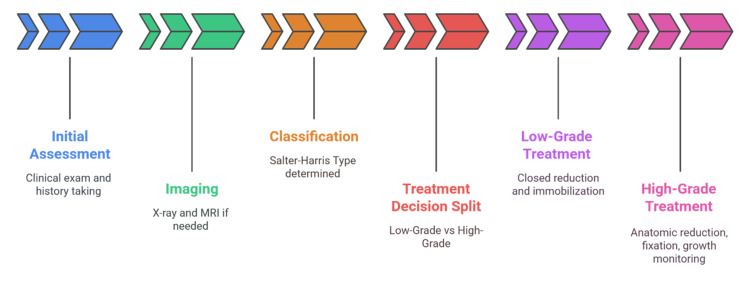
Pediatric Fracture Management Pathway Image Credit: Sanjay P

The use of patient-reported outcome measures is also becoming a requirement in order to reflect the overall effect of the fractures on everyday life and recovery [12]. New priorities are the creation of validated functional outcome measures that are specific to pediatric fractures and uniform follow-up plans to monitor the growth disturbances over time. Figure 2 illustrates the stepwise approach to pediatric physical fractures, from initial assessment and imaging through classification and treatment selection.

Scoliosis and spinal deformities

The term scoliosis refers to a lateral deviation in the spine that reaches more than 10 degrees along with rotation of the vertebrae [1]. The condition occurs in idiopathic, congenital, and neuromuscular forms and has different etiological and management options [35]. Almost 80% of the cases are related to adolescent idiopathic scoliosis (AIS), which is thought to be caused by multifactorial genetic, hormonal, and neuromuscular factors, but a specific causative pathway has not been clearly defined [3]. The neuromuscular scoliosis is usually linked to cerebral palsy (CP) or muscular dystrophy caused by the imbalance of muscle and gradual spinal instability [36].

The most important thing is to detect it early, when it can be corrected, and nothing serious will happen to a child because of the deformity. The old ways of screening were using visual inspection and the forward-bending test, which were not accurate in assessment; newer technology has changed this [15]. Surface-mapping methods involving projection of contour lines, such as moire topography, are also able to detect small asymmetries imperceptible to the naked eye, but their popularisation has been hampered by technical complexities and by the existence of simpler methods such as scoliometer screening [6]. Moreover, the EOS imaging systems that produce low-dose biplanar radiographs, with the concomitant three-dimensional reconstructions, have greatly improved the accuracy of Cobb angle measurement and evaluation of rotational deformities [27]. Nevertheless, EOS imaging is not widely available in most environments, and this is based on the cost of equipment and infrastructure.

There is a treatment decision based on the magnitude of the curve, skeletal maturity, and risk of progression. Skeletally immature patients with curves measuring 25-45 degrees are advised to be braced in a bid to ensure that they do not progress to surgical levels [8]. The recent findings substantiate the effectiveness of rigid thoracolumbosacral orthoses, especially those worn at least 18 hours a day, with a maximum success rate of 72% in preventing curve progressions [9]. However, bracing protocols are not always well-adhered to, and there are reported rates as low as 50% in some series, so patient education and support are important. In cases of curves that are greater than 45-50 degrees or in cases of rapidly progressive deformities, surgery is recommended to restore coronal and sagittal balance and maintain pulmonary functions [37].

**Figure 3:**
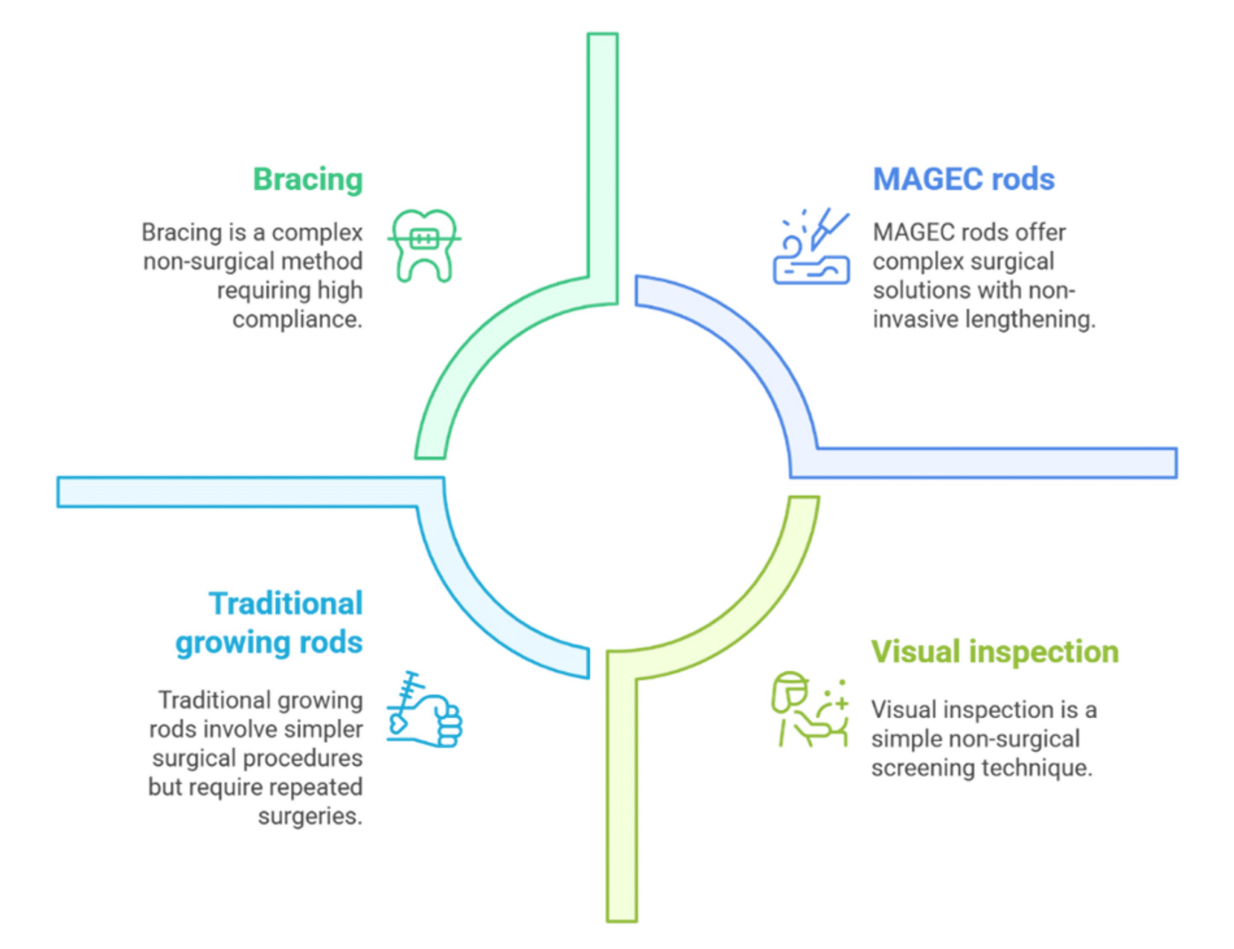
Comparative Overview of Scoliosis Screening and Treatment Approaches Image Credit: Sanjay P MAGEC: magnetic expansion control

Growth-friendly instrumentation has transformed the management of early-onset scoliosis. The old growing rods necessitated frequent surgeries every six months to lengthen them, and the anesthesia dose was repeated, and there was a risk of infection [11]. Surgical morbidity and hospital visits have been minimized by the introduction of magnetic expansion control (MAGEC) rods that allow non-invasive lengthening by an external remote controller [38]. The recent series has shown a 40-50% correction of the mean curve and a reduction of 50% of surgical procedures in comparison with the traditional growing rods [13]. Nevertheless, some complications, including rod breakage and metallosis, can be observed in 10-15% of cases and need to be thoroughly monitored in the long-term perspective. Additionally, there is limited long-term data on the preservation of spinal growth, as well as pulmonary function, and some studies have expressed concerns about unanticipated distraction failure. The future directions are the improvement of the implant material and methods to reduce mechanical failure and increase the efficacy of growth modulation [39]. Multicenter prospective studies are required to establish the best protocols for the selection of implants, the increased intervals, and the time of final fusion. Figure 3 shows four important ways of scoliosis treatment, and their non-surgical screening procedures are visual inspection and bracing, whereas surgical procedures are traditional growing rods and the MAGEC rods.

Legg-Calvé-Perthes disease

The Legg-Calve-Perthes disease is an idiopathic epiphyseal osteonecrosis of the femoral head of a child between the ages of four and eight years [15].

Pathophysiology: It is characterized by temporary impairment of the blood supply of the femoral head, which causes ischemic necrosis, revascularization, and remodeling [40]. Radiographic stages of disease evolution as per Waldenstrom classification are initial, fragmentation, reossification, and healing, whereas the prognosis based on the extent of lateral femoral head involvement in the process of fragmentation is stratified as per the lateral pillar classification [7]. Staging is very important since it informs the management of containment and surgery. The best way is to diagnose it early so as to implement containment measures in order to maintain the sphericity of the femoral head. The conventional radiographs are the main mode of diagnosis, which could be normal during the early phase of ischemia [18]. MRI is a sensitive means of early detection, as it shows marrow edema, subchondral fractures, and perfusion deficits before they are radiographically apparent [41]. The degree of avascular involvement can be measured by the use of dynamic contrast-enhanced sequences, which are used in prognosis and treatment planning [20]. Nevertheless, access to high-level imaging is low in numerous contexts, which is one of the factors that leads to the late diagnosis and the more advanced stage of the disease at its initial presentation.

The treatment options also rely on the stage of the disease, age of the patient, and extent of the femoral head affected. The children under the age of six years and with less than 50% involvement of the head may be treated with observation and activity modification since the immature skeleton has remodeling abilities [21]. The containment techniques, such as Petrie casting and abduction bracing, are used to position the femoral head back into the acetabulum during reossification [42]. Proximal femoral varus osteotomy or Salter innominate osteotomy are considered as surgical containment procedures and are usually reserved for older children who have severe lateral pillar collapse [23]. Although they are widely used, the comparative effectiveness of such surgical procedures compared to nonoperative management is controversial because of the absence of randomized controlled trials, and the majority of the data are based on observational cohorts. The prognosis depends upon the severity of involvement of the epiphysis, the age of the patient, and sphericity during healing. Better results are found when treatment is in younger patients and in patients with preservation of lateral pillar height. The long-term studies revealed that up to 60% of patients with widespread collapse developed symptomatic osteoarthritis within 30 years of the diagnosis [4]. Thus, early treatment and close follow-ups are necessary to make the best out of long-term hip usage and postpone degenerative processes. The future research directions are the investigation of regenerative biologic therapies, including bone marrow aspirate concentrate and growth factor injections, and hip preservation procedures, to delay or prevent joint degeneration [43]. Future studies that include standardized functional outcomes measures and imaging biomarkers will be relevant to establish the best treatment pathways and the patients who will have the best response to the new treatments.

Slipped capital femoral epiphysis (SCFE)

SCFE has been characterized as posterior and inferior displacement of the femoral head relative to the neck across the growth plate [33]. SCFE normally happens during the adolescent growth spurt, with the highest number of cases between ages 11 and 14 [29]. A close connection with obesity has been demonstrated in epidemiological studies, as this raises shear stress throughout the hypertrophic physis and predisposes to slip [35]. Other risk factors include endocrinopathies such as hypothyroidism, growth hormone deficiency, and renal osteodystrophy [26]. SCFE is termed stable when the patient can ambulate, even with crutches, and unstable when ambulation is not possible. Up to 50% of unstable slips carry a high risk of avascular necrosis [27].

Prompt diagnosis and stabilization are essential to avoid further displacement and ischemic injury. Anteroposterior and frog-leg lateral radiographs are part of the standard diagnostic workup, with the frog-leg view being the most sensitive [32]. Improvements in MRI have enabled earlier detection of physeal widening and signal changes reflective of pre-slip, which can guide prophylactic treatment [28]. However, ideal criteria for pre-slip diagnosis remain debatable, and standardized MRI protocols have not been universally adopted [31]. Treatment objectives include stabilizing the physis, preventing progression, and reducing complications [30]. Single-screw in situ fixation remains the gold standard for stable slips, with high rates of union and satisfactory functional outcomes [34]. Reported progression of slip after in situ fixation is under 5% when surgery is performed promptly, highlighting the importance of early intervention [33]. The timing and indication for prophylactic pinning of the contralateral hip remain controversial. Some authors recommend prophylactic fixation in high-risk patients, such as those younger than 12 or with endocrinopathies, due to contralateral slip rates approaching 40% [35]. Others advocate close monitoring with serial radiographs to avoid unnecessary surgery [27]. Family-shared decision-making is essential, as no high-level evidence conclusively favors one approach over the other [26].

The most difficult type of management is unstable or severe slips, which present a high risk of osteonecrosis [30]. It has been replaced by the modified Dunn procedure, which is surgical hip dislocation and realignment of the epiphyses, and has gained popularity in specialized centres [36]. In addition to the modified Dunn technique, the PARSCH procedure, which involves controlled open reduction with subcapital realignment without full surgical hip dislocation, has gained attention as an alternative approach for unstable slips. This method aims to restore proximal femoral alignment while minimizing vascular compromise, and early clinical reports suggest promising outcomes with potentially lower rates of avascular necrosis, although robust long-term comparative studies are still lacking. In the recent series, better proximal femoral anatomy restoration and avascular necrosis rates of 10-20% have been reported compared with greater than 40% with in situ fixation in severe unstable slips [36]. Nevertheless, this method is technically challenging and comes with increased risk of operation, and thus it needs expert teams of surgeons [44]. Most of the evidence in favor of the superiority of the modified Dunn procedure is based on single-center cohort studies, and further validation should be established before the wide application.

The long-term results are influenced by the severity of the slip, the early treatment, and the deformity that remains [38]. Early and mild slips normally produce excellent functioning and a low risk of arthritis, but late and severe slips are likely to result in chronic pain and early degenerative changes [45]. Residual femoroacetabular impingement may also emerge even after successful fixation, and thus, correctional procedures may be necessary. Future research will address the need to perfect the MRI-based risk stratification, the development of growth plate-sparing implants, and long-term comparative studies of prophylactic contralateral fixation against observation [39]. Moreover, multicentric registries and standardized reporting of functional outcomes will be useful to make evidence-based recommendations and enhance the consistency of care. Table 2 presents the key points of SCFE, such as its definition, epidemiology, risk factors, classification, diagnosis, management, and future directions.

**Table 2:** Overview of Slipped Capital Femoral Epiphysis

Aspect	Details	Diagnosis and Imaging	Treatment Options	Prognosis and Future Directions	Reference
Definition	Posterior and inferior displacement of the femoral head epiphysis through the growth plate	Radiographs (AP and frog-leg lateral views); frog-leg lateral views are most sensitive	Stable slips: in situ fixation with a single cannulated screw	Mild slips treated early: excellent outcomes; severe slips: risk of early arthritis	[26]
Epidemiology	Occurs during adolescent growth spurt (ages 11–14); strong association with obesity; contralateral slip risk up to 40% in high-risk patients.	MRI detects early physeal widening and pre-slip changes	Unstable slips: modified Dunn procedure increasingly used in specialized centers; higher technical demands	Residual femoroacetabular impingement is possible even after fixation	[7]
Risk Factors	Obesity, hypothyroidism, growth hormone deficiency, renal osteodystrophy	MRI improves early detection; criteria for pre-slip diagnosis remain debated	Timing of prophylactic contralateral pinning is controversial; shared decision-making is recommended	Research: growth plate–sparing implants, MRI risk stratification, standardized functional outcome reporting	[18]
Classification	Stable (can ambulate) vs. unstable (cannot ambulate); unstable slips have ~50% avascular necrosis risk.	Early imaging is critical to prevent progression and ischemic injury	In situ fixation yields <5% slip progression when performed promptly	Multicenter registries are needed to validate treatment strategies and improve the evidence base	[31]
Challenges	Late diagnosis increases deformity and complications; there is a lack of consensus on pre-slip criteria and prophylactic fixation indications.	Standard radiographs are essential; MRI is helpful for subtle slips	Modified Dunn procedure: better restoration of anatomy but higher operative risk; requires experienced surgical teams	Long-term studies are needed to define optimal protocols and outcomes	[25]

Limb length discrepancy and deformity correction

LLD is a major clinical dilemma because it has many causes and may lead to a long-term effect on musculoskeletal well-being [12]. Although differences of less than 2 cm in most cases are well tolerated, increasing ones may sequentially undermine gait mechanics and joint alignment [13]. The effects of untreated LLD are not confined to biomechanics; as such, compensatory pelvic obliquity and secondary degenerative alterations may develop in the long term [11].

The emergence of motorized intramedullary lengthening systems has transformed the surgical technology in the recent past [17]. This transition is reflected in the PRECICE nail that provides better patient-friendly options as opposed to the standard circular external fixators [14]. However, indications should be highly individualized, considering such aspects as skeletal maturity, bone quality, and risk profiles of the patient [16]. The limitation of costs and disparities in access also complicate the choice of treatment, which makes multidisciplinary planning and counseling of patients important [18].

Another significant change in preoperative planning and intraoperative accuracy is the increasing presence of computer-aided platforms [19]. The tools enable surgeons to simulate a complex deformity correction and restore the mechanical axis with a high level of fidelity [15]. Nevertheless, this is not enough to carry out widespread adoption because it is necessary to provide consistent training pathways and standardized protocols to guarantee a reproducible outcome [21].

The effective treatment of LLD always involves close cooperation of orthopedic surgeons, physiotherapists, and rehabilitation specialists [20]. Although the technical part of lengthening is the most important, the rehabilitation after the surgery and close follow-ups are also decisive in the long-term success [22]. In the future, studies dedicated to smart implants that have their embedded sensors and automatic feedback systems have the potential to make the process even safer and more accurate. Table 3 shows the key points of the LLD, such as its causes, the way to diagnose it, the methods of its treatment, and the priorities of future studies.

**Table 3:** Overview of Limb Length Discrepancy: Etiology, Diagnosis, Management, and Future Directions

Aspect	Details	Diagnostic Approaches	Treatment Strategies	Future Directions	Reference
Definition	Difference in limb length due to congenital or acquired causes	Clinical exam and standing long-leg radiographs	Intramedullary lengthening (PRECICE nails), external fixation (Ilizarov)	Prospective trials comparing systems, smart implants, and improving access to advanced technologies	[11]
Etiology	Congenital: fibular hemimelia, congenital femoral deficiency; Acquired: physeal injury, infection, trauma	Long-leg alignment radiographs quantify mechanical axis deviation	PRECICE nails: magnetically driven distraction; fewer infections, less pain	Standardization of preoperative planning protocols and surgeon training	[3]
Impact if Untreated	>2 cm discrepancy can cause gait compensation, pelvic obliquity, and degenerative joint disease	Computer-assisted analysis: Taylor Spatial Frame, Bone Ninja	Average length gains: 4–6 cm; complication rates 5–15% (implant failure, regenerate fracture)	Development of smart monitoring systems and sensors for real-time distraction tracking	[25]
Preoperative Planning	Software simulation of lengthening and deformity correction improves accuracy and reduces intraoperative guesswork	Nutritional and physical optimization are recommended	Careful patient selection is essential; it is contraindicated in very young children with narrow canals or poor bone stock	Multidisciplinary protocols integrating surgical, rehabilitation, and nutritional optimization	[17]
Rehabilitation	Focus on maintaining joint mobility, promoting regenerative bone consolidation, and supporting gait retraining	Regular radiographic monitoring during the distraction and consolidation phases	Postoperative physiotherapy is essential to prevent stiffness and optimize function	Expanding the availability and affordability of advanced limb reconstruction techniques	[7]
Challenges	High cost, limited availability in some regions, variable surgeon experience	Close monitoring during lengthening is required to avoid distraction loss or implant failure	Multidisciplinary team including pediatric orthopedic surgeons, rehabilitation specialists, and physical therapists	Building multicenter registries and outcome tracking databases	[33]

CP is the leading cause of motor disability in childhood, resulting from nonprogressive harm to the growing brain [33]. Orthopedic manifestations are varied, with the interaction of spasticity, muscle weakness, and abnormal growth [28]. The common problems are muscle contractures, hip subluxation and dislocation, scoliosis, and foot defects [46]. The prevalence of hip displacement among children with CP reaches up to one-third of all patients, where it is more prevalent in non-ambulatory individuals and those belonging to Gross Motor Function Classification System (GMFCS) levels IV and V [47]. Progressive hip subluxation may cause pain, hygiene problems, and functional impairments if not treated.

New surveillance procedures of the hip focus on early detection of progressive migration prior to the development of pain and fixed deformity [30]. Recent consensus guidelines suggest frequent radiographic follow-up (every 12-24 months of age, depending on the level of GMFCS [36]. With early detection, reconstructive osteotomies or guided growth procedures can be performed in time to prevent loss of function. Gait analysis has proved to be an indispensable instrument in surgical planning for ambulatory children with CP. Multilevel procedures are targeted at quantifying kinematic and kinetic abnormalities with three-dimensional motion capture systems [32]. Gait laboratory-guided surgery has been shown to enhance energy expenditure, gait symmetry, and patient-reported outcomes compared to traditional clinical evaluation [25]. Access to gait labs is, however, still limited in most regions, and unified protocols for interpretation are still under development [37].

Spasticity management forms the cornerstone of nonoperative care. Temporary muscle overactivity is reduced with botulinum toxin injections, delaying surgical treatment in younger children [34]. Dorsal rhizotomy, which decreases afferent lower limb input, has demonstrated lasting effects on spasticity and gait in properly selected patients [29]. The selection criteria typically include ambulatory children with spastic diplegia as the major component and minimal dystonia [47]. Muscle-tendon lengthening, femoral osteotomy, and spinal fusion for scoliosis remain essential orthopedic procedures to address fixed contractures and skeletal deformity [35].

Functional outcomes of multilevel surgical techniques applied as single-event multilevel surgery (SEMLS) are superior to staged interventions, for ambulatory patients at least, though high-level evidence remains limited [26]. Long-term prognosis depends on timely intervention, family engagement, and coordinated multidisciplinary care [28]. Research priorities include developing predictive models to inform surgical timing, advanced imaging biomarkers of muscle pathology, and optimizing rehabilitation strategies to maintain functional gains [32]. Additionally, studies incorporating standardized functional outcome measures and health-related quality-of-life assessments will be important to support evidence-based practice [33].

Bone and joint infections in children

The most frequent bone and joint infection in the pediatric population is acute hematogenous osteomyelitis and septic arthritis [38]. Osteomyelitis normally occurs as the hematogenous implantation of metaphyseal circulation, which is promoted by the slow flow of growing bone [39]. *Staphylococcus aureus* is the most common pathogen, with methicillin-resistant strains (MRSA) of which are becoming an increasingly high percentage of cases in most areas [40]. *Kingella kingae *has proven to be a significant pathogen of septic arthritis, particularly in young children below four years [48]. According to recent reports, *Kingella *has been estimated to cause up to 40% of culture-negative cases of septic arthritis among this age group. Clinical manifestation involves local pain, swelling, fever, and reduced use of limbs. Laboratory results of increased C-reactive protein, erythrocyte sedimentation rate, and leukocytosis are favorable but not specific [42]. MRI has now become the imaging modality of choice, and it is more sensitive, with a sensitivity of more than 90% to identify early osteomyelitis and soft tissue involvement [43]. The latest modes of diagnosis, such as polymerase chain reaction tests, have enhanced the identification of these pathogens, especially *Kingella*, which is hard to culture [44]. Nevertheless, uniform procedures of molecular diagnostics are only under development, and they are not so widespread in most environments.

There has been a management transformation to shorter intravenous antibiotic treatment and subsequent oral treatment. A recent research study backed the idea of three to five days of parenteral therapy in uncomplicated osteomyelitis, on the condition that the clinical improvement should be observed and inflammatory markers should be normalized [49]. The method is linked with comparable cure rates and reduced hospital stay rates as compared to long-term intravenous treatment. In septic arthritis, surgical drainage is also necessary as soon as possible to avoid destruction of cartilage [46]. The use of arthroscopic lavage is gaining popularity in treating hip and shoulder infections because of the enhanced visibility and low morbidity [47]. Conversely, joints such as the knee and ankle are still subjected to open arthrotomy in most centers. According to the consensus guidelines, the antibiotic treatment of uncomplicated osteomyelitis should take three to four weeks and septic arthritis two to three weeks [50]. The more complex cases involving abscesses, chronic infection, or immunocompromise are treated with longer courses [41]. Although there is an increasing agreement, there is still controversy on the best antibiotic regimen and duration of its use, particularly in MRSA infection and children younger than three months. The orthopedic surgeons, infectious disease specialists, and radiologists working multidisciplinarily achieve the best results and reduce sequelae [30]. The priorities of future research involve prospective trials that would compare antibiotic strategies, studies that would assess the role of novel imaging modalities in monitoring the response to treatment, and the creation of biomarkers to predict the failure of a treatment.

Limitations and future directions

Although a significant amount of improvement has been made in the pediatric orthopedic practice, there are a number of issues that limit the available evidence. The limited number of high-quality randomised controlled trials due to ethical restrictions, limited sample size, and variability in disease presentation has led to the development of high variability in the diagnostic criteria and treatment modality between institutions and geographical areas. As an example, brace thresholds in AIS and length of antibiotics in osteomyelitis vary significantly between centers and are frequently dependent on local experience and resources. The durability of interventions and late complications cannot be judged easily by long-term follow-up studies since patient attrition is often a problem, as well as changing surgical practices. Moreover, numerous studies are based on radiographic evaluation or clinician-reported outcomes that cannot be compared with each other or allow for understanding the applicability of results to patient-centered care. The need to have validated outcome measures that will measure functional abilities, participation, and quality of life with time still exists. This review is organized by major clinical conditions rather than regional anatomy to provide a concise, clinically focused overview of the pediatric orthopedic disorders with the highest global prevalence and practice impact.

In the future, it is important to focus on the incorporation of artificial intelligence and machine learning technologies in order to improve the accuracy of diagnosis, predict treatment outcomes, and plan surgery. The possibility of patient-specific implants and 3D-printed surgical guides has the promise of increased precision and personalization of complex reconstructions. Genetic and molecular therapies Genetic and molecular therapies are in preclinical or early phases of development and may one day allow disease-modifying therapies to be developed to treat congenital disorders of the bone, including osteogenesis imperfecta and skeletal dysplasias. Additionally, the inclusion of patient-reported outcomes and health-related quality-of-life measures in clinical trials will allow us to make sure that the emerging treatments improve daily functioning in a significant way. Finally, although telemedicine platforms may help increase access to follow-up care and rehabilitation in underserved environments, issues of digital infrastructure constraints, clinician education, and data safety will have to be addressed with care in order to facilitate the equitable application and long-term sustainability. Multicenter research networks and international registries will also be required to develop strong evidence and to align care standards in a variety of practice settings.

## Conclusions

The range of congenital and acquired orthopedic disorders of children is wide, and their impact on growth and functioning, as well as the quality of life, may be significant. The development of diagnostic imaging, minimally invasive surgery, and treatment algorithms has dramatically changed the outcomes of diseases such as dysplasia of the hip, clubfoot, scoliosis, fractures, and neuromuscular syndromes. Nevertheless, there are remaining issues of variability of bracing protocols, lack of high-quality comparative data, and high disparities in access to specialized care, especially in low-resource settings where delays often lead to preventable disability.

In the future, pediatric orthopedics will have the advantage of incorporating new technologies, such as artificial intelligence-based diagnostic prediction systems, enhanced imaging biomarkers to stratify early disease, and patient-specific interventions such as 3D-printed implants and magnetically controlled growth rods. Meanwhile, it will be essential to spread the practice of using standardized outcome measures that are reported by patients to make sure that innovations can lead to significant changes in everyday performance and well-being. The areas of future research should be big prospective trials, the development of predictive models to inform individual treatment plans, and strategies to establish multidisciplinary networks that enhance equity in care delivery. Long-term investment and global cooperation are going to be needed to evolve evidence-based practice and maximize musculoskeletal well-being and lifelong potential of children affected by it across the globe.
